# Diffusion Tensor Imaging of Brain Abnormalities Induced by Prenatal Exposure to Radiation in Rodents

**DOI:** 10.1371/journal.pone.0107368

**Published:** 2014-09-09

**Authors:** Shigeyoshi Saito, Kazuhiko Sawada, Miwa Hirose, Yuki Mori, Yoshichika Yoshioka, Kenya Murase

**Affiliations:** 1 Department of Medical Physics and Engineering, Division of Medical Technology and Science, Faculty of Health Science, Graduate School of Medicine, Osaka University, Osaka, Japan; 2 Faculty of Medical and Health Sciences, Tsukuba International University, Tsuchiura, Japan; 3 Biofunctional Imaging Lab, Immunology Frontier Research Center (WPI-IFReC), Osaka University, Osaka, Japan; 4 Center for Information and Neural Networks (CiNet), National Institute of Information and Communications Technology, and Osaka University, Osaka, Japan; Radboud University, The Netherlands

## Abstract

We assessed brain abnormalities in rats exposed prenatally to radiation (X-rays) using magnetic resonance imaging (MRI) and histological experiments. Pregnant rats were divided into 4 groups: the control group (*n* = 3) and 3 groups that were exposed to different radiation doses (0.5, 1.0, or 1.5 Gy; *n* = 3 each). Brain abnormalities were assessed in 32 neonatal male rats (8 per group). Ex vivo T_2_-weighted imaging and diffusion tensor imaging (DTI) were performed using 11.7-T MRI. The expression of markers of myelin production (Kluver–Barrera staining, KB), nonpyramidal cells (calbindin-D28k staining, CaBP), and pyramidal cells (staining of the nonphosphorylated heavy-chain neurofilament SMI-32) were histologically evaluated. Decreased brain volume, increased ventricle volume, and thinner cortices were observed by MRI in irradiated rats. However, no abnormalities in the cortical 6-layered structure were observed via KB staining in radiation-exposed rats. The DTI color-coded map revealed a dose-dependent reduction in the anisotropic signal (vertical direction), which did not represent reduced numbers of pyramidal cells; rather, it indicated a signal reduction relative to the vertical direction because of low nerve cell density in the entire cortex. We conclude that DTI and histological experiments are useful tools for assessing cortical and hippocampal abnormalities after prenatal exposure to radiation in rats.

## Introduction

Many studies of survivors of prenatal exposure to the atomic bombings of Hiroshima and Nagasaki have shown that exposure to ionizing radiation during pregnancy has harmful effects on the development of the human central nervous system (CNS) [1], [2], [3]. Animal embryos are sensitive to radiation, and irradiation of a fertilized egg with as little as 0.1 Gy is lethal. In addition, exposure of an embryo to radiation can cause various diseases, such as hydrocephalus and microcephaly [3]. These CNS alterations have been observed in animal models after prenatal X-ray irradiation at day 15 of pregnancy in rats and at day 13 of pregnancy in mice [4]. Moreover, brain abnormalities, such as hippocampal atrophy and ventricular dilatation, have been observed in rodents after exposure to radiation [5], [6]. The atrophy of the corpus callosum in mice is maximal when irradiation takes places at day 13 of gestation [7]. Several reports based on in vivo magnetic resonance imaging (MRI) have shown that CNS damage, such as vessel malformation and ventricular dilatation, was present in rats exposed to radiation prenatally [8] [9].

Anisotropic water diffusion in neural fibers is more prominent in white matter (WM)-rich tissues [10], [11], [12]. The anisotropy of water diffusion has become an important and widely used tool for studying CNS connectivity [13] and pathologies [14], [15]. Various structural components of the WM tissue may contribute to the restricted diffusion observed when water diffusion is measured perpendicular to the long axis of the WM fibers. These components include myelin sheaths, axonal membranes, microtubules, and neurofilaments [12]. In recent years, several specific animal models characterized by demyelination and dysmyelination have been used to study the effect of myelin on the diffusion characteristics of WM tissue. Song et al. used DTI to study diffusion anisotropy in the case of dysmyelination in shiverer mice. They found increased radial diffusivity without changes in axial diffusivity [16]. DTI performed in studies by Nair et al. [17] and Tyszka et al. [18] also demonstrated that lack of myelin in shiverer mice affects water diffusion anisotropy. Moreover, DTI is a useful tool for examining and quantifying WM and gray matter (GM) microstructures in animal models of CNS disease.

Our goal was to use DTI to assess the brain abnormalities and changes in cellular organization induced by prenatal exposure to radiation, focusing on the cortex and hippocampus. In addition, a histological analysis was performed to evaluate the markers of myelin production (Kluver–Barrera staining, KB), nonpyramidal cells (calbindin-D28k staining, CaBP), and pyramidal cells (SMI-32 staining) in this model.

## Materials and Methods

### Animal Procedures

Pregnant female Sprague–Dawley (SD) rats (n = 12; weight, 284±23.9 g; Japan SLC, Hamamatsu, Japan) were allowed to rest for 1 week before the experiment. The animals had free access to food and water and were kept under standard laboratory conditions of 22–23°C, ∼50% humidity, and a 12/12-h light/dark cycle. The 12 pregnant female rats were divided into 4 groups, comprising 1 control (*n* = 3) and 3 radiation-exposed (*n* = 9) groups. The radiation-exposed groups underwent a single exposure to whole-body X-ray irradiation at a dose of 0.5 (*n* = 3), 1.0 (*n* = 3), or 1.5 Gy (*n* = 3), respectively, on day 15 of pregnancy [5] [19]. The X-irradiation was delivered at a dose rate of 0.88 Gy/min (120 kV, 15 mA with a 1-mm-thick aluminum filter; Rigaku Radioflex 350 X-Ray Generator, Osaka, Japan). After birth, 8 neonatal male rats were selected at random from each group, for a total of 32 neonatal rats.

These male SD rats were divided into 4 groups for all experiments: (1) 4-week-old control rats (n = 8; weight, 123.8±8.3 g), (2) 4-week-old rats exposed prenatally to 0.5 Gy of radiation (n = 8; weight, 102.3±10.1 g), (3) 4-week-old rats exposed prenatally to 1.0 Gy (n = 8; weight, 91.4±7.6 g), and (4) 4-week-old rats exposed prenatally to 1.5 Gy (n = 8; weight, 73.9±7.5 g). Regardless of the prenatal conditions (control or radiation exposure), all neonatal rats were kept with their mothers in regular light/dark cycles until the MRI experiments were performed at 4 weeks after birth. MRI experiments were performed in all 8 rats per group, and histological experiments were performed in 4 rats per group.

### Ethics Statement

This study was carried out in strict accordance with the recommendations of the Guide for the Care and Use of Laboratory Animals of the National Institutes of Health. The protocol was approved by the Committee on the Ethics of Animal Experiments at the University of Osaka (Permit Number: 23-040-0).

### MRI Measurements

Before MRI scanning, all 4-week-old rats were euthanized via an overdose of pentobarbital (Dainippon Sumitomo Pharma Co., Ltd., Osaka, Japan) and prepared for MRI experiments by perfusion-fixation with saline containing heparin, followed by 7.5% paraformaldehyde, for 1 month. All brains were extracted. MRI experiments were performed on an 11.7-T MRI scanner (Bruker Biospin, Ettlingen, Germany) and a volume radiofrequency (RF) coil with an inner diameter of 25 mm was used for transmission and reception (m2m Imaging Corp., Cleveland, Ohio, USA). The center of the imaging slices was carefully set at bregma −0.36 mm with reference to the rat brain atlas of Paxinos and Watson [20]. Transaxial multislice T_2_-weighted MR imaging (T_2_WI; rapid acquisition with relaxation enhancement [RARE]; TR/TE, 3000/70 ms; slice thickness, 1.0 mm; matrix, 256×256; field of view, 25.6×25.6 mm^2^; number of repetitions, 4; number of slices, 16; no slice gap) was conducted. Diffusion imaging was performed on all rats, using SE multi-shot echo-planar imaging (TR, 6000 ms; TE, 33 ms; slice thickness, 1 mm, field of view, 25.6×25.6 mm^2^; matrix, 128×128; slice orientation, transaxial; number of repetitions, 6; number of slices, 16; no slice gap; diffusion directions, 6; b-values, 1000 s/mm^2^). The DTI reconstruction and fractional anisotropy (FA) calculation were performed with the manufacturer-supplied software (Paravision 5.1, Bruker). The DTI fiber orientation was determined with reference to the rat brain atlas of Paxinos and Watson [20]. Brain volume was evaluated manually using 16 sequential slices (18). The ventricle volume was defined using the following procedures of ImageJ (Ver. 1.40 g, National Institutes of Health, Bethesda, MD, USA): 1) the “threshold” plug-in for generating binary images, and 2) the “measure” plug-in for measuring ventricle volume. Cortical, corpus callosum (CC), and external capsule (EC) thickness were evaluated manually in a slice approximately −0.36 mm posterior to the bregma, based on the rat brain atlas of Paxinos and Watson [20], using the ImageJ “measurement” plug-in.

### Histological Experiments

The brains were removed from the skull, and were embedded in Optimal Cutting Temperature (OCT) compound, with reference to the rat brain atlas of Paxinos and Watson [20]. The coronal sections (thickness, 40 µm) were made to correspond to the MR images (−0.36 mm of bregma) by a Retratome (REM-700; Yamato Koki Industrial, Asaka, Japan) with a refrigeration unit (Electro Freeze MC-802A, Yamato Kohki Industrial Co., Ltd.). Sections were immersed in 3% H_2_O_2_ in PBS containing 0.1% Triton X-100 to inactivate endogenous peroxidase, and were then incubated with the Antigen Retrieval Reagent UNIVERSAL (R&D Systems, Inc., MN, and USA) for 30 min at 90°C. After washing with PBS, sections were incubated with a rabbit anti-calbindin D-28k (CaBP) polyclonal antibody (1∶10,000; Swant, Switzerland), or a mouse anti-SMI-32 monoclonal antibody (1∶1,000, Covance, Princeton, NJ, USA), containing 10% normal goat serum at 4°C overnight. After incubation, the sections were rinsed with PBS and incubated with biotinylated anti-rabbit IgG or biotinylated anti-rat IgG. The immunoreactive products were visualized with a Vectastain ABC elite kit (Vector Labs., Inc., Burlingame, CA) using 0.01% 3,3′-diaminobenzidine tetrachloride (Sigma) in 0.03% H_2_O_2_ as a chromogen. KB staining for myelin was carried out [21]. DTI and KB, CaBP, and SMI-32 staining were compared visually to identify anatomical structures, such as the cortical 6-layered structure (I–VI), corpus callosum, cingulum, external capsule, and anterior commissure, using the rat brain atlas [20]. We defined the 6 layers of the cerebral cortex based on cytoarchitecture, with reference to a previous paper [22] that shows the laminar structures of the primary somatosensory cortex of rats that had received different doses of X-irradiation on gestational day 15.

### MRI Data Analysis and Statistics

All statistical analyses were performed using Prism 5 (Version 5, GraphPad Software, USA). One-way analysis of variance (ANOVA) with Dunnett was used to compare changes against control animals in the FA value, brain volume, ventricle volume, and cortical thickness across groups of radiation-exposed animals. Correlation analysis between radiation doses and MRI were performed using Pearson product-moment correlation analysis. *P*<0.05 was considered significant.

## Results

**Figure 1:**
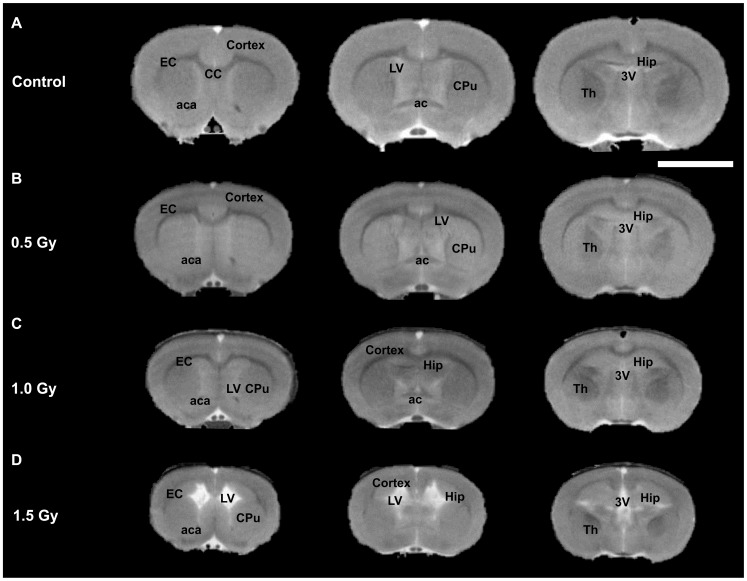
T_2_-weighted MRI (T_2_WI) results. Each row: 3 T_2_W images representing a consecutive series of brain slices in a typical rat from one of the experimental groups. (A) The first row shows images from a typical control rat. (B) The second-row images are from a typical 0.5-Gy–irradiated rat. (C) The third-row images are from a typical 1.0-Gy–irradiated rat. (D) The bottom-row images are from a typical 1.5-Gy–irradiated rat. The brain size of the animals in the radiation-exposed groups is clearly smaller than that of the control rats. CC, corpus callosum; EC, external capsule; aca, anterior commissure anterior part; LV, lateral ventricle; 3V, 3rd ventricle; CPu, caudate-putamen; Hip, hippocampus; TH, thalamus; ac, anterior commissure; white bar, 5 mm.

Typical T_2_WI results for all groups are shown in Figure 1A–1D. Table 1 shows that the brain volume of 4-week-old rats exposed to 0.5, 1.0, or 1.5 Gy of X-radiation prenatally was smaller than that of the control rats. In contrast, the lateral-plus-3^rd^ ventricle volume of rats at the same age exposed prenatally to the same three levels of radiation dose was larger than that of the control rats. The cortices were also thinner than those of the control rats. The CC and EC of rats exposed to 0.5 and 1.0 Gy of radiation prenatally were thinner than those of the control rats. These results show decreased brain volume, increased ventricle volume, thinner cortex, thinner CC, and thinner EC in irradiated rats and all effects were dose dependent.

**Table 1:** Morphological measurements of the brains of radiation-exposed and control rats using MRI.

Radiation dose	Control	0.5 Gy	1.0 Gy	1.5 Gy
Brain volume (mm^3^)	1756±78.9	1321.9±72.4***	1062.2±109.8***	828.2±42.8***
Ventricle volume (mm^3^)	1.7±0.5	3.4±1.0	6.4±1.4***	27.1±3.3 ***
Cortex thickness (µm)	1662.5±89.8	1452.5±143.6**	963.0±120.9***	829.3±136.2***
CC thickness (µm)	627.2±118.0	536.8±89.3	477.7±61.3**	N.D. (Split)
EC thickness (µm)	545.3±76.2	501.7±63.9	315.6±35.2***	257.3±35.6***

The brain size of 4-week-old radiation-exposed rats was smaller than that of the 4-week-old control rats. In contrast, the volume of the lateral-plus-3rd ventricles of 4-week-old rats exposed to 1.0 or 1.5 Gy of radiation was larger than that of the control rats. The thicknesses of the cortex, CC, and EC of 4-week-old rats exposed to 1.0 or 1.5 Gy of radiation were less than those of the control rats. Significant thinning of the cortex was also found for the smallest dose, vs. control, *P*<0.05;

**, vs. control, *P*<0.01;

***, vs. control, *P*<0.001

**Figure 2:**
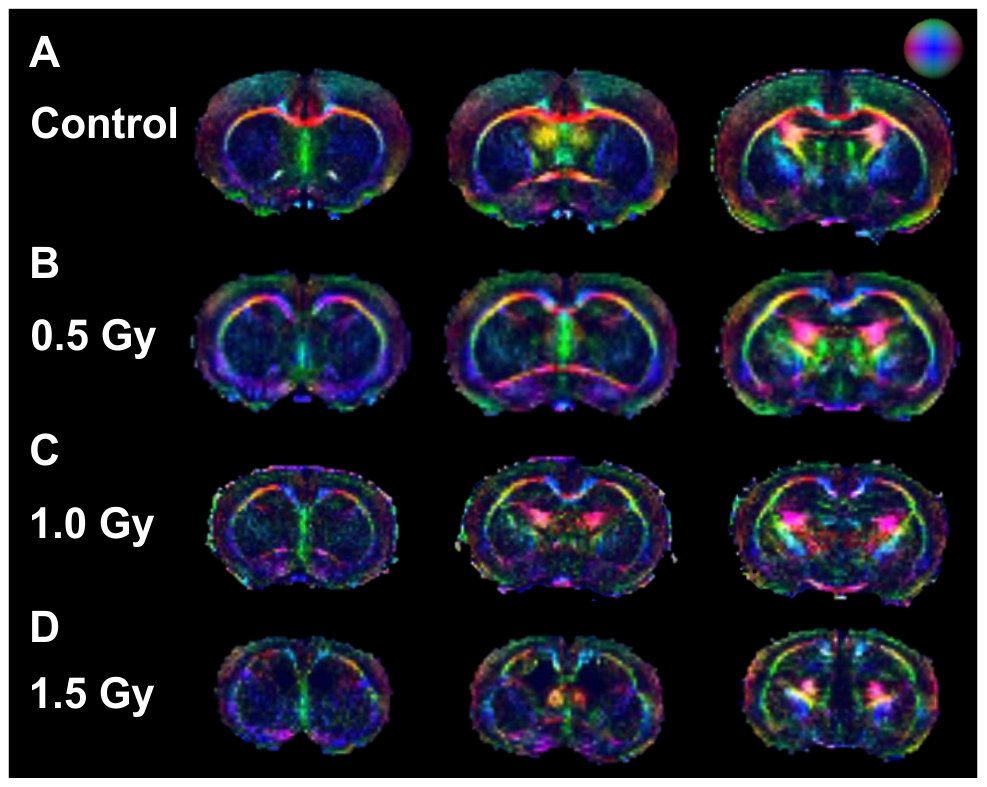
Typical diffusion tensor imaging (DTI) MRI results. Each row: DTI 3-color maps representing a consecutive series of brain slices, in a typical rat from one of the experimental groups. Color maps of tracked fibers were based on the extracted primary direction (red was used for the x-direction, green for the y-direction, and blue for the z-direction). A–D; rows have the same meaning as in Figure 1.

**Figure 3:**
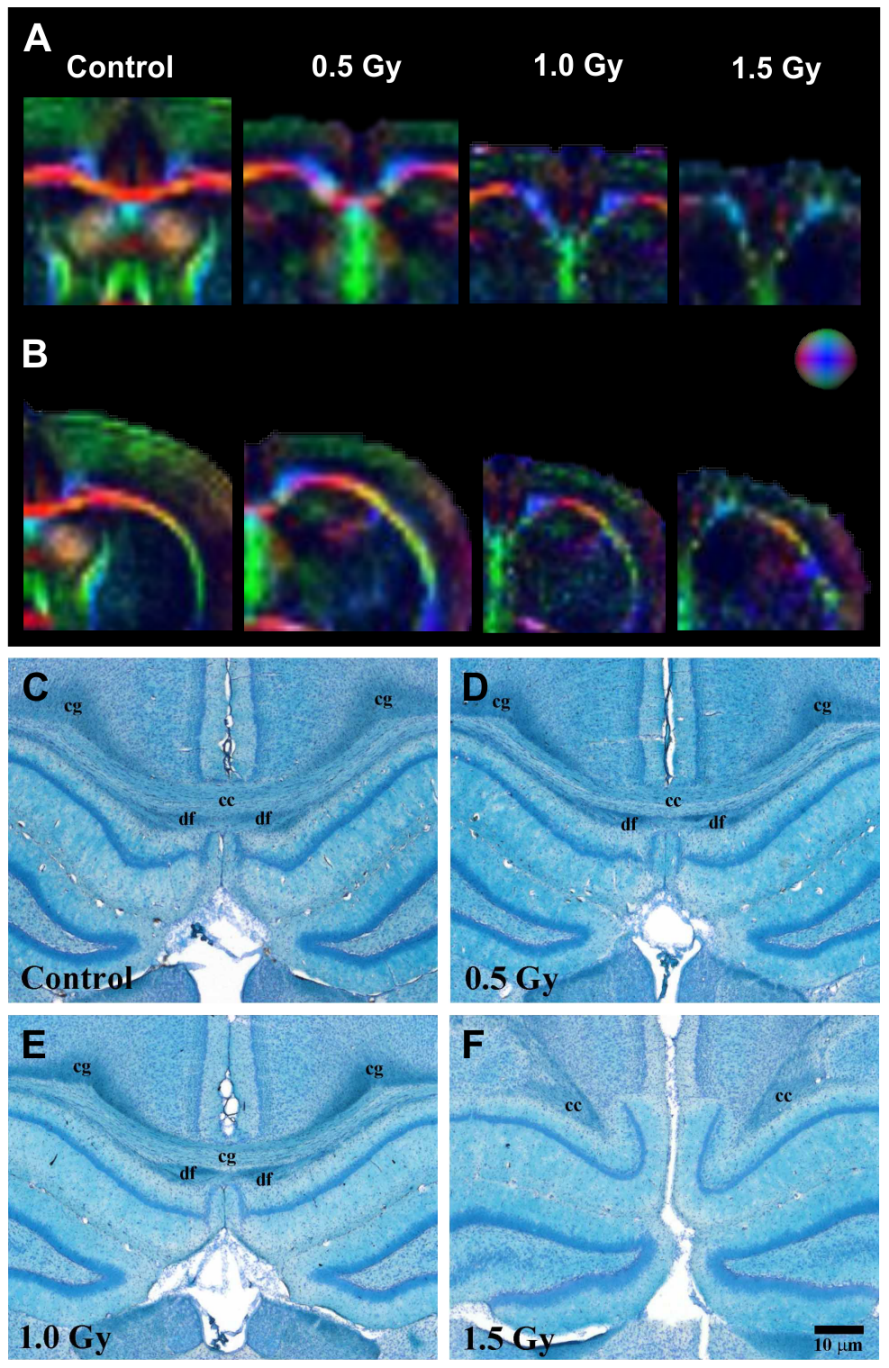
Typical DTI and KB-staining images. (A) The first column of color-scale images shows typical coronal DTI rat brain images at CC. (B) The second column shows typical coronal DTI rat brain images at EC. (C) The third column shows typical KB staining images around CC. Rows have the same meaning as in previous figures. EC, external capsule; df, dorsal fornix; Cg, cingulum.

Figure 2A–2D shows typical DTI color maps of the brain for all groups. The changes in DTI signal in WM areas such as the CC and EC are shown in detail in Figure 3. The corpus callosum, which is characterized by axial (transverse) fiber orientation, appears predominantly in red, whereas the cingulum, in which the fibers run in the front–rear direction, appears in blue in Figure 2A–2D. In addition, the cortical mantle, which is characterized by horizontal fiber orientation, appears predominantly in green (Figures 2A, 2B, 3A, and 3B). The comparison of the DTI color maps of the control group (Figure 2A) with those of the radiation-exposed groups (Figure 2B–2D) revealed an obvious decrease in green-color fiber densities in the somatosensory (S1) cortex of radiation-exposed rats. In control animals and in rats exposed to 0.5 Gy (Figure 2B) or 1.0 Gy (Figure 2C) of radiation prenatally, fibers of the CC were identified using KB staining (Figure 3C–3E). However, the histological images of the 1.5-Gy radiation-exposed group showed an absence of fiber organization in the CC (Figure 3F). The same result was obtained based on DTI images (Figure 3A), which provided evidence of fiber disorganization in the corpus callosum of 1.5 Gy radiation-exposed rats. Table 2 shows that the FA values in the cortex of 4-week-old rats exposed to 1.0 Gy or 1.5 Gy were smaller than those of the control rats were. In addition, the FA values in the CC of rats exposed to 1.0 Gy were smaller than those of control rats. FA values were not measured at the CC in 1.5-Gy–irradiated animals because the axial fibers were split. Moreover, the FA values in the EC of rats exposed to 0.5, 1.0, or 1.5 Gy were all smaller than those of the control rats.

**Table 2:** FA values.

Radiation dose	Control	0.5 Gy	1.0 Gy	1.5 Gy
Cortex	0.25±0.01	0.24±0.02	0.23±0.02*	0.17±0.02***
Corpus callosum (CC)	0.51±0.04	0.48±0.04	0.40±0.03***	N.D. (Split)
External capsule (EC)	0.59±0.04	0.52±0.04*	0.44±0.05***	0.37±0.06***

The FA (fractional anisotropy) values in the cortex and CC of 4-week-old rats exposed to 1.0 or 1.5 Gy of radiation prenatally were smaller than those of control rats. At 1.5 Gy, FA values were not measured at the CC because the axial fibers were split. FA values in the EC were significantly smaller than those of the control rats at all 3 exposures (0.5, 1.0, or 1.5 Gy).

*, vs. control, *P*<0.01;

***, vs. control, *P*<0.001

**Figure 4:**
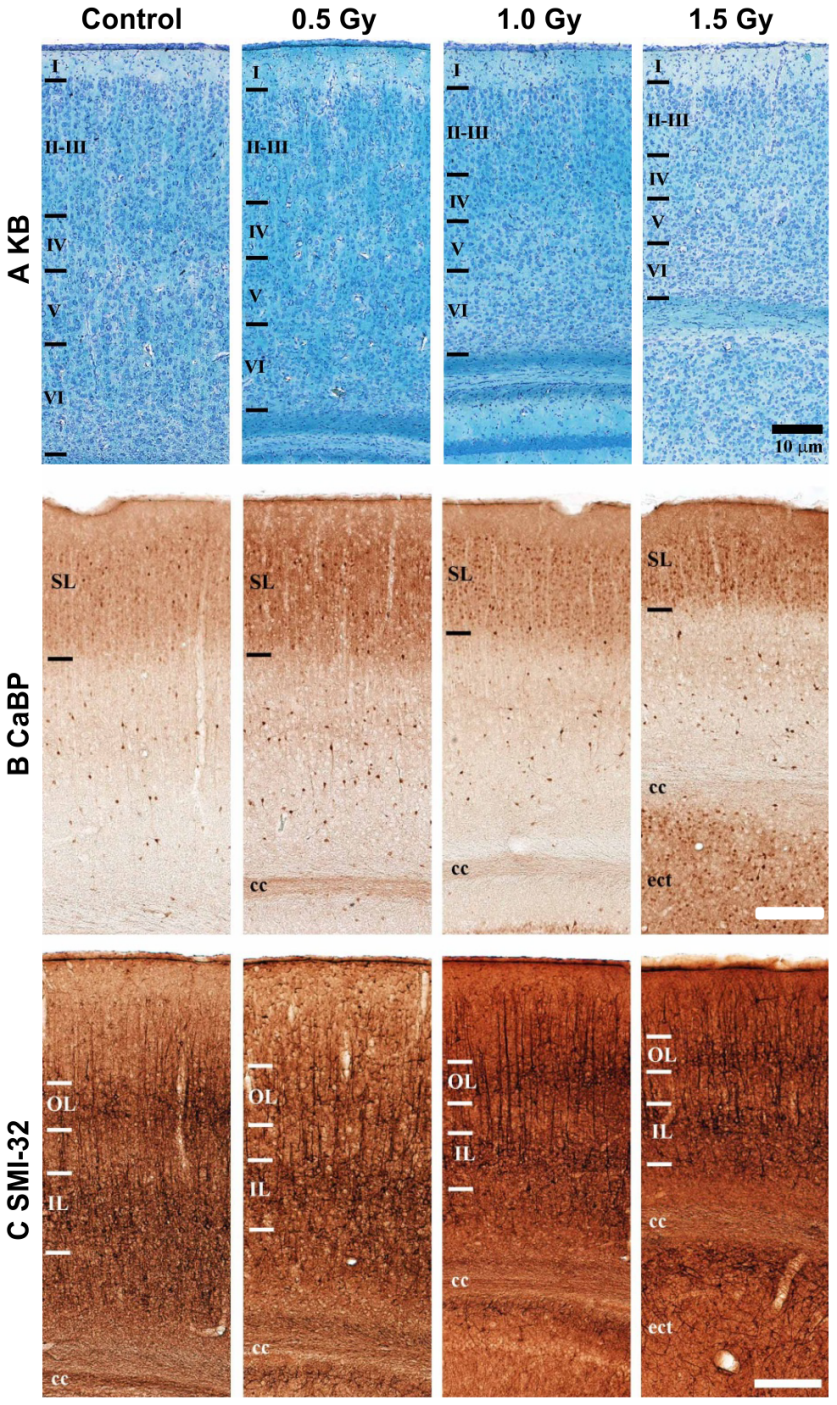
Histological analyses performed using KB, CaBP, and SMI-32 staining in sections of cortex. (A) The first column shows typical coronal KB images of 6-layered cortex. (B) The second column shows typical coronal CaBP images at the same positions as those used for KB staining. (C) The third column shows typical SMI-32 images at the same positions as those used for KB staining. Rows, progressively higher X-ray dose, 0 Gy at top; white and black bars, 10 µm; Cg, cingulum; df, dorsal fornix; SL, superficial layer; OL, outer layer; IL; inner layer.

Histological sections around the S1 cortex stained with KB, CaBP, and SMI-32 are shown at similar magnifications in Figure 4. KB staining is helpful for visualizing white matter and can be seen as a bright blue color (Figure 4A). We identified the 6 layers of the cortex in all control and radiation-exposed rats (Figure 4A). In the S1 cortex, CaBP-positive cells were observed in neuropil of layers I–III (SL) and in nonpyramidal cells throughout the cortex (Figure 4B). SMI-32 staining is used to visualize neuronal cell bodies, dendrites, and some thick axons in the central and peripheral nervous systems; however, thin axons are not observed using this technique (Figure 4C). Both the outer layer (OL) and the inner layer (IL) became thinner, and layer-specific abnormalities were absent in the cortices of radiation-exposed rats (Figure 4C).

**Figure 5:**
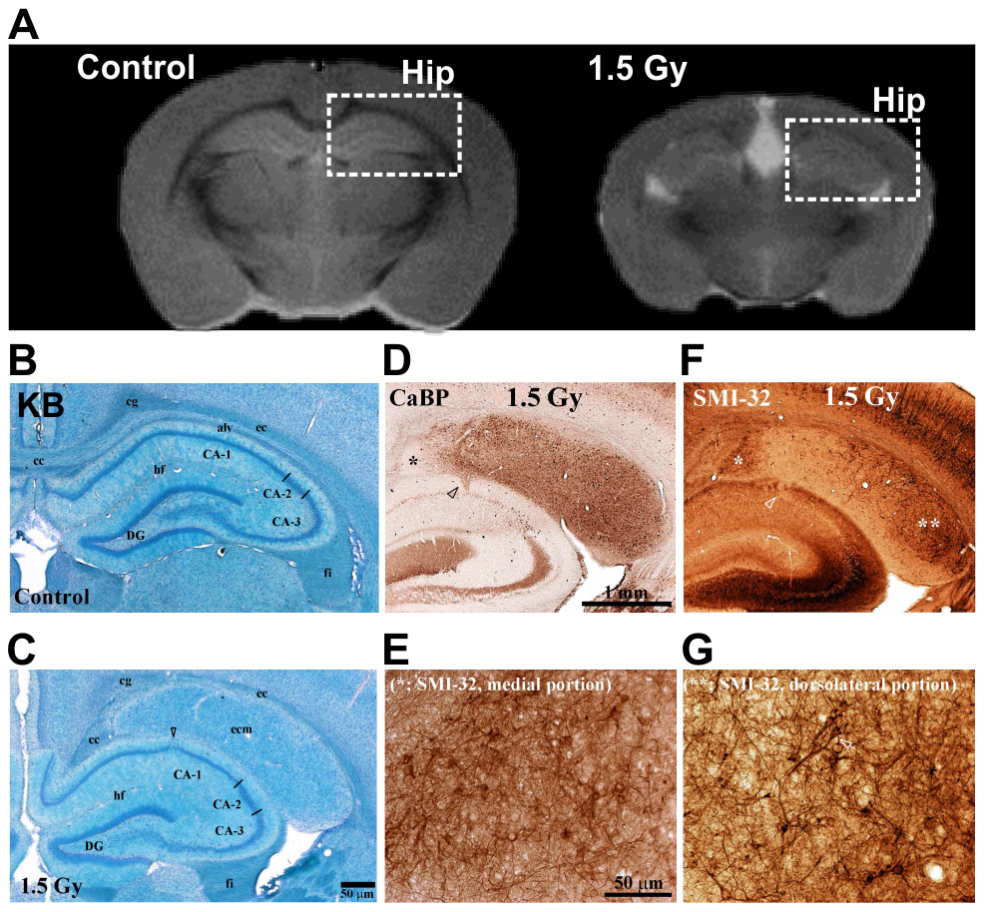
Histological analyses performed using KB, CaBP, and SMI-32 staining in sections of the hippocampus. (A) Typical images around the hippocampus in sections from control and 1.5-Gy–irradiated rat brains. The upper gray-scale images show typical control and 1.5-Gy–irradiated rat T_2_WI results. B, C; Typical control (B) and 1.5-Gy–irradiated (C) rat KB images in sections of the hippocampus. D, E; Typical coronal CaBP images in sections of the hippocampus at low and high magnification. F, G; Typical SMI-32 images in sections of the hippocampus. Hip, hippocampus; alv, alveus of the hippocampus; hf, hippocampal fissure; ecm, ectopic neuron mass; f, fimbria of the hippocampus; DG, dentate gyrus; black bars, 50 µm in C and E, and 1 mm in D.

Figure 5 shows the results of MRI (Figure 5A) and histological experiments using KB, CaBP, and SMI-32 staining (Figures 5B–5G) in hippocampus sections. The present study showed prominent hippocampal atrophy in the brains of rats exposed prenatally to radiation (Figure 5A, 1.5 Gy). Ectopic neurons were observed beneath the cerebral cortex in 1.5-Gy–irradiated rats, (Figure 5C–5F). The neuropil was mostly CaBP-positive, with the exception of the inner portion (* in Figure 5D). SMI-32-positive neurons were observed in the dorsolateral part of the CaBP-positive areas (** in Figure 5F). Cells with a pyramidal-cell-like morphology (Figure 5, white arrow) were also observed among the dorsal outer ectopic SMI-32-positive cells, with random directions of extension of dendrites (Figure 5C).

**Figure 6:**
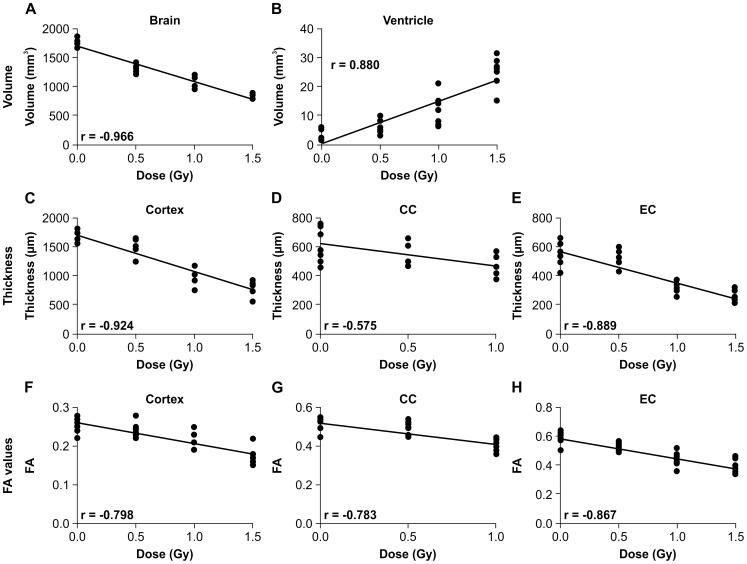
Correlation analysis: correlations of MRI-determined brain volume, ventricle volume, brain substructure thicknesses, and brain substructure FA (fractional anisotropy) values with radiation dose were evaluated. A, B; Correlation between radiation dose (0.5, 1.0, or 1.5 Gy) and brain volume (A) and ventricle volume (B). C, D, E; Correlation between dose and thickness of cortex (C), thickness of corpus callosum (D), and thickness of external capsule (E). F, G, H; Correlation between dose and FA values of cortex (F), corpus callosum (G), and external capsule (H).

Figure 6 shows the correlation between irradiated dose and MRI-determined parameters. A robust negative correlation was identified for brain volume against radiation dose (r = −0.966, *P*<0.0001, Figure 6A). A robust positive correlation was identified for ventricle volume against radiation dose (r = 0.880, *P*<0.0001, Figure 6B). A robust negative correlation was identified for cortical thickness against radiation dose (r = −0.924, *P*<0.0001, Figure 6C). On the other hand, negative correlations against radiation dose were found for thickness of CC (r = −0.575, *P* = 0.004, Figure. 6D) and thickness of EC (r = −0.889, *P*<0.0001, Figure 6E). In the DTI study, a negative correlation was found against radiation dose for FA in the cortex (r = −0.798, *P*<0.0001, Figure 6F), CC (r = −0.783, *P* = 0.005, Figure 6G), and EC (r = −0.867, *P*<0.0001, Figure 6H).

## Discussion

This report evaluated the presence of cortical and hippocampal abnormalities in rats prenatally exposed to radiation, using T_2_WI, DTI, and histological staining for myelin (KB), nonpyramidal cells (CaBP), and pyramidal cell (SMI-32).

### Brain Morphological Evaluation Using MRI

The effects of irradiation on the developing rodent CNS have been discussed for many years. Many macroscopic and microscopic analyses have reported that rodent brains exposed prenatally developed microcephaly, ectopic gray matter, reduction of cortical thickness, abnormal neuronal migration, and disturbance of synaptogenesis [8], [23]. Kitamura et al. reported that most neural cells targeted by the apoptosis process triggered in mouse brains by prenatal ionizing irradiation were neurons [24]. Apoptosis was shown to target neurons and oligodendrocytes in the dentate gyrus of the hippocampus of adult mice [25]. Gobbel et al. showed that neurons are more susceptible to apoptosis by X-irradiation than are astrocytes [6]. Those authors stated that much of the neuronal apoptosis observed in mice irradiated at embryonic days 15 and 17 was associated with thinner cerebral cortex, decreased the size of the whole brain, and a reduction in brain and whole-body weight postnatally.

Miki et al. reported that the brain weight of rats X-ray irradiated prenatally was significantly lower than that of control rats. Similarly, at 4 weeks after birth, the brain volume of rats exposed to X-ray irradiation prenatally was dose-dependently smaller than that of control rats, as shown by MRI. This observation of brain volume as evaluated using MRI is in agreement with the results of a previous histological study [26]. In addition, the present study showed a prominent dilatation of the cerebral ventricles and spaces around the whole brain, as well as hippocampal atrophy, in the brains of rats exposed prenatally to radiation. Previous studies using animal models and histological examination demonstrated the presence of congenital malformations in radiation-exposed rat brains [4], [27], [28]. Moreover, Miki et al. found histological and morphological abnormalities in the hippocampus, such as hippocampal atrophy, after prenatal X-ray irradiation [27], [28]. It has been shown that radiation-induced congenital malformations, such as hydrocephalus, are responsible for the dilatation of the cerebral ventricles and disruption of vascular endothelial cells [27], [28]. Ectopic neurons were observed beneath the cerebral cortex in rats exposed to 1.5 Gy prenatally in our study. Sun et al. identified the types and 3-dimensional distributions of neocortical ectopias after prenatal exposure to X-irradiation, as assessed using histological examination and computer-based reconstruction techniques [29]. The present MRI observations are in agreement with the results of previous studies in this regard.

A previous work noninvasively characterized the regional postnatal radiation-induced developmental alterations observed in the mouse brain, using longitudinal MRI [30]. The authors of that study characterized radiation-sensitive brain regions based on changes in volume and growth rate after irradiation at 7 Gy [30]. Their MRI and histology results were consistent with previously published work [31] [32] and provide an additional spatiotemporal map of postnatal radiation-induced developmental changes. Those authors observed a radiation-induced loss of WM volume on MRI, which was associated with a demyelination seen histologically. They found that many GM regions were also affected, leading to a significant reduction in overall brain volume in irradiated animals. Cell loss within the neurogenic niches of the subgranular zone of the dentate gyrus and of the subventricular zone was apparent histologically. In our study, we identified prenatal radiation-induced developmental alterations in the rat brain. The exposure age was different between the previous work (at 2.5 weeks after birth) [30] and our study (embryonic day 15). However, our MRI-based brain morphological observations in rats that were exposed to radiation prenatally are in agreement with the results of previous studies of rats exposed postnatally.

### Pathological Correlates of White and Gray Matter Signal Alterations Seen in MRI

The comparison of the DTI color maps of the control group with those of the radiation-exposed groups revealed an obvious decrease in green fiber densities in the radiation-exposed rat cortex. The thickness of layers I–III (SL) in radiation-exposed rats was dose-dependently smaller than that of control rats. However, a 6-layered structure was observed in all control and irradiated rats (0.5–1.5 Gy), as assessed by KB staining. Similarly, CaBP-positive cells were observed in the neuropil of SL and in pyramidal cells throughout the cortex in all rats. In addition, SMI-32-positive cells were observed at the cell body and dendrite areas of pyramidal cells, which allowed a clear distinction between layer III (OL) and layer V (IL) in control and irradiated rats. However, IL also became dose-dependently thinner, so that layer-specific abnormalities were not seen in the cerebral layered structure of radiation-exposed rats. The FA values in the cortex of 4-week-old rats exposed to 1.0 or 1.5 Gy of radiation were dose-dependently smaller than those of the control rats. These observations were in agreement with the findings from KB staining. Moreover, a dose-dependent reduction in the anisotropic signal (green) in the vertical direction was observed in DTI, which was not mainly due to abnormal axonal fiber extension. A 6-layered structure was observed in all control and irradiated rats (0.5–1.5 Gy) using KB, CaBP, and SMI-32 staining. These observations did not represent an absence of pyramidal cells; rather, they reflected the reduction in the signal relative to the vertical direction because of a decrease in a real nerve cell density in the entire cortex. In addition, the FA values of rats that were exposed to X-ray irradiation prenatally were dose-dependently smaller than those of control rats, as shown by DTI in the CC and EC. Abnormalities of CC and EC WM structures were observed in all irradiated rats (0.5–1.5 Gy), as assessed using KB staining. We suggest that these abnormalities in the WM of irradiated rat brains detected by KB staining are consistent with the reduction of FA values assessed by DTI.

Prominent hippocampal atrophy was observed in the brains of rats that were exposed to radiation prenatally. Sun et al. also detected neocortical ectopias after prenatal X-irradiation, as assessed using histological examination [29]. The same ectopic neurons were observed beneath the cerebral cortex in rats exposed to 1.5 Gy of radiation prenatally in our study, as assessed using both MRI and KB staining. These neuropils were mostly CaBP-positive cells, with the exception of an inner portion in the hippocampus. These results suggest a migration failure of late-generated neurons after prenatal irradiation. SMI-32-positive neurons were observed in the dorsolateral part of the ectopic CaBP-positive areas. SMI-32-positive cells in late-generated neurons colocalized with outer dorsal ectopic cells. It has been shown that ectopic negative cells were localized from the center to the ventral side of the cell masses. SMI-32-positive cells located within areas containing inner ectopic cells were considered the pyramidal cells of layer V, because they were located in CaBP-negative areas. However, we were not able to determine whether these were in pyramidal cell layer V based on shape and size. In addition, we also observed the presence of cells with a pyramidal-like morphology among the SMI-32-positive cells located within areas containing outer ectopic cells, with a random direction of dendrite extension. This is consistent with the lack of particular anisotropy in ectopic cells in DTI maps.

## Conclusions

This study found that decreased brain volume, increased ventricle volume, and reduced cortical and white matter thickness in irradiated rats can be assessed using MRI. Rats that were irradiated at embryonic day 15 day exhibited postnatal changes such as thinner cerebral cortex, decreased size of the whole brain, and increased ventricle volume. Comparison of the DTI color maps obtained here revealed decreased horizontal color fiber densities in the S1 cortex of radiation-exposed rats. However, no abnormalities in the cortical 6-layered structure were observed in any of the radiation-exposed rats. Moreover, ectopic neurons were observed only in the hippocampus (beneath the cerebral cortex) of rats exposed to 1.5 Gy of radiation prenatally. DTI is a useful tool for examining and quantifying cortical and hippocampal abnormalities in model rats exposed to radiation prenatally.
